# Predictive performance of eLIFT for liver inflammation and fibrosis in chronic liver diseases

**DOI:** 10.7150/ijms.62386

**Published:** 2021-08-27

**Authors:** Zongguo Yang, Xin Ma, Xinlan Zhou, Dan Huang, Yanbing Wang, Xiufen Li, Wei Lu, Zhanqing Zhang, Rongrong Ding

**Affiliations:** 1 Department of Hepatobiliary Medicine, Shanghai Public Health Clinical Center, Fudan University, Shanghai 201508, China.; 2 Department of Integrative Medicine, Shanghai Public Health Clinical Center, Fudan University, Shanghai 201508, China.; 3 Department of Ultrasound, Shanghai Public Health Clinical Center, Fudan University, Shanghai 201508, China.

**Keywords:** eLIFT, GPR, liver fibrosis, chronic liver disease

## Abstract

**Objective:** The easy liver fibrosis test (eLIFT) is a novel predictor of liver fibrosis in chronic liver disease (CLD). This study aimed to evaluate the predictive value of the eLIFT for liver inflammation and fibrosis in CLD patients.

**Methods:** We enrolled 1125 patients with CLD who underwent liver biopsy. The predictive accuracy for liver inflammation and fibrosis of the eLIFT was assessed and compared to that of the aspartate aminotransferase-to-platelet ratio index (APRI), fibrosis-4 score (FIB-4), and gamma-glutamyl transpeptidase-to-platelet ratio (GPR) by ROC (Receiver Operating Characteristic) analysis and decision curve analysis (DCA).

**Results:** The areas under the ROC curves (AUROCs) of the eLIFT for assessing liver inflammation G ≥ 2 and G ≥ 3 were 0.77 (0.75-0.80) and 0.81 (0.79-0.84), with cut-offs of 8.0 and 11.0, respectively. The AUROCs of the eLIFT for predicting fibrosis stages S ≥ 2 and S4 were 0.72 (0.70-0.76) and 0.76 (0.72-0.80), with cut-offs of 9.0 and 10.0, respectively. In discriminating G≥2 inflammation, the AUROC of the eLIFT was better than that of the FIB-4, with no difference compared with the GPR, but lower than that of the APRI. When discriminating G≥3 inflammation, the AUROC of the eLIFT was comparable to that of the APRI and GPR but superior to that of the FIB-4. There were no significant differences between the four indexes for predicting S≥2 and S4.

**Conclusion:** The eLIFT is a potentially useful noninvasive predictor of liver inflammation and fibrosis in patients with CLD.

## Introduction

Chronic liver disease (CLD), of which liver fibrosis is a common consequence, is a major public health problem with high morbidity and mortality worldwide. In 2015, viral hepatitis caused 1.34 million deaths 1. The incidence rate of alcoholic liver disease and nonalcoholic fatty liver disease is also rising 2-4. Fibrosis progression contributed significantly to an increased risk of cirrhosis. If not properly managed, liver functional impairment, subsequent structural deformation, and haemodynamic deterioration may lead to portal hypertension-related complications and an increase in the incidence of liver cancer 5. Therefore, the early detection of liver fibrosis is very important in the treatment of CLD to prevent irreversible damage.

At present, liver biopsy is still considered the gold standard procedure for accurately assessing liver histological scores. However, the wide clinical application of this procedure is restricted due to its drawbacks, such as invasiveness, patient discomfort, sampling error, potential risk of complications, and interobserver variability 6,7. Clinical practice requires simple procedures or noninvasive and simple methods to diagnose liver inflammation and fibrosis 8. Transient elastography (TE) has been introduced as a noninvasive, highly reproducible technique for the assessment of liver fibrosis, which may reduce the need for liver biopsy 9-11. However, some drawbacks, such as expensive equipment and lack of trained operators, limit the clinical application of TE, especially in resource-limited environments. Therefore, many studies have concentrated on the development of simple and practical serum noninvasive markers that are more accessible to the majority of the public 12.

The World Health Organization (WHO) has recommended serum biomarkers, including the aspartate aminotransferase to platelet ratio index (APRI) and four factor-based fibrosis index (FIB-4), as alternative methods for liver biopsy 13, 14. However, the performances of the APRI and FIB-4 for the evaluation of liver fibrosis are still controversial 15, 16. The gamma-glutamyl transpeptidase to platelet ratio (GPR) is more accurate than the APRI and FIB-4 in estimating liver fibrosis in West African cohorts with CHB (chronic hepatitis B), but it was not superior to the APRI and FIB-4 in a French cohort 14. Other studies have also not observed advantages of the GPR 17, 18.

Based on 2503 patients with CLD, Boursier et al. 19 developed a novel panel, the easy Liver Fibrosis Test (eLIFT), which was used to diagnosis liver fibrosis and cirrhosis. Compared to other blood markers, the eLIFT is easily calculated, as it was equivalent to a sum of points attributed to age, sex, GGT, AST, platelet count and prothrombin time. However, its use in the diagnosis of inflammation and fibrosis in CLD patients with different aetiologies is limited.

The aim of this study was to compare the clinical significance of the eLIFT, APRI, FIB-4, and GPR for staging liver inflammation and fibrosis in CLD patients with different aetiologies using the histopathology of liver biopsies as the reference standard.

## Materials and Methods

### Study Population

A total of 1125 consecutive patients with chronic liver disease who underwent percutaneous liver biopsy at Shanghai Public Health Clinical Center, Fudan University, from January 2015 to December 2019 were retrospectively studied. All the patients were > 18 years old. Inclusion criteria were diagnosis of treatment-naïve chronic viral hepatitis (hepatitis B or C), alcoholic liver disease, nonalcoholic fatty liver, or autoimmune hepatitis, and being off potential transaminase-lowing agents for at least 2 weeks prior to routine laboratory tests. The exclusion criteria were hepatocellular carcinoma, HIV coinfection, antiviral treatment history, decompensated cirrhosis, inadequate liver biopsy samples (<1.5 cm), and pregnancy.

### Liver biopsy

Percutaneous liver biopsy was performed using a 16 G needle under ultrasound guidance. Liver samples with a minimum length of 1.5 cm and at least 6 complete portal tracts were considered suitable for liver histological scoring. Liver histology was analysed by two experienced pathologists who were blinded to other clinical and laboratory data and classified according to the Scheuer scoring system 20: G 0-4 and S 0-4.

### Routine laboratory parameters

Fasting blood samples were obtained within a week of liver biopsy. Platelets and other blood cells were counted using a Sysmex-XT 4000i automated haematology analyser. Prothrombin time and other coagulation indexes were measured using a STAR Max automatic coagulation analyser. Alanine transaminase (ALT), aspartate aminotransferase (AST), alkaline phosphatase (ALP), γ-glutamyl transferase (GGT), and other serum biochemical parameters were measured using an Architectc16000 automatic biochemical analysis system.

### Formulas

The formulas for the eLIFT, APRI, FIB-4, and GPR are described here. The eLIFT score is the sum of assigned values for age, sex, AST, GGT, platelet counts, and prothrombin time(%): age (years): < 40 = 0, ≥ 40 = 3; sex: female = 0, male = 1; AST (IU/L): < 35 = 0, 35-69 = 2, ≥ 70 = 4; GGT (IU/L): < 35 = 0, 35-89 = 1, ≥ 90 =2; platelet counts (10^9^/L): ≥ 250 = 0, 170-249 = 1, < 170 = 4; prothrombin time (%): ≥ 97 = 0, 84-96 = 2, < 84 =4. The APRI is calculated as (AST (U/L)/ULN of AST)/platelet count (10^9^/L) × 100. The FIB-4 is calculated as (age (years) × AST (U/L))/(platelet count (10^9^/L) × (ALT (U/L)) ^1/2^). The GPR is calculated as (GGT (U/L)/ULN of GGT)/platelet count (10^9^/L) × 100.

### Statistical analysis

Statistical analysis was performed using IBM SPSS Statistics version 26.0 (SPSS Inc., Chicago, USA) and R 4.0.2 (http://www.R-project.org). Continuous variables are given as the median (range) and compared using the independent Mann-Whitney test. Categorical variables are given as proportions and compared by the chi-squared test. Correlations were evaluated by Spear's correlation coefficient for continuous variables. The performances of serum models for predicting liver histological scores were assessed by receiver operating characteristic (ROC) curve analyses and area under the ROC curves (AUROCs). The Delong Z test was used to compare the AUROCs of the serum models. Decision curve analysis (DCA) was used to further evaluate predictive performance. A two-sided P<0.05 was considered statistically significant.

## Results

### Baseline characteristics of study patients

One thousand one hundred twenty-five patients with chronic liver disease were enrolled in our study. The baseline clinical characteristics of the study cohort are described in **Table 1**. Of them, the patients had a median age of 37 (30-46) years, and the majority were male (65.2%). The majority of patients suffered from chronic hepatitis B (CHB) (75.8%). The distribution of liver inflammatory activities was 585 (52.0%) patients with G0-1 and 540 (48.0%) with G2-3. The distribution of fibrosis stages was 583 (51.8%) patients with S0-1 and 542 (48.2%) with S2-4. Generally, compared with patients in G0-1, patients in G2-3 had higher ALT, AST, and GGT levels, eLIFT scores, APRIs, FIB-4s, and GPRs, but lower platelet counts and prothrombin activities. Similar trends in these noninvasive markers were also observed in patients with fibrosis S2-4.

### Serum markers and liver histological scores

**Figure 1:**
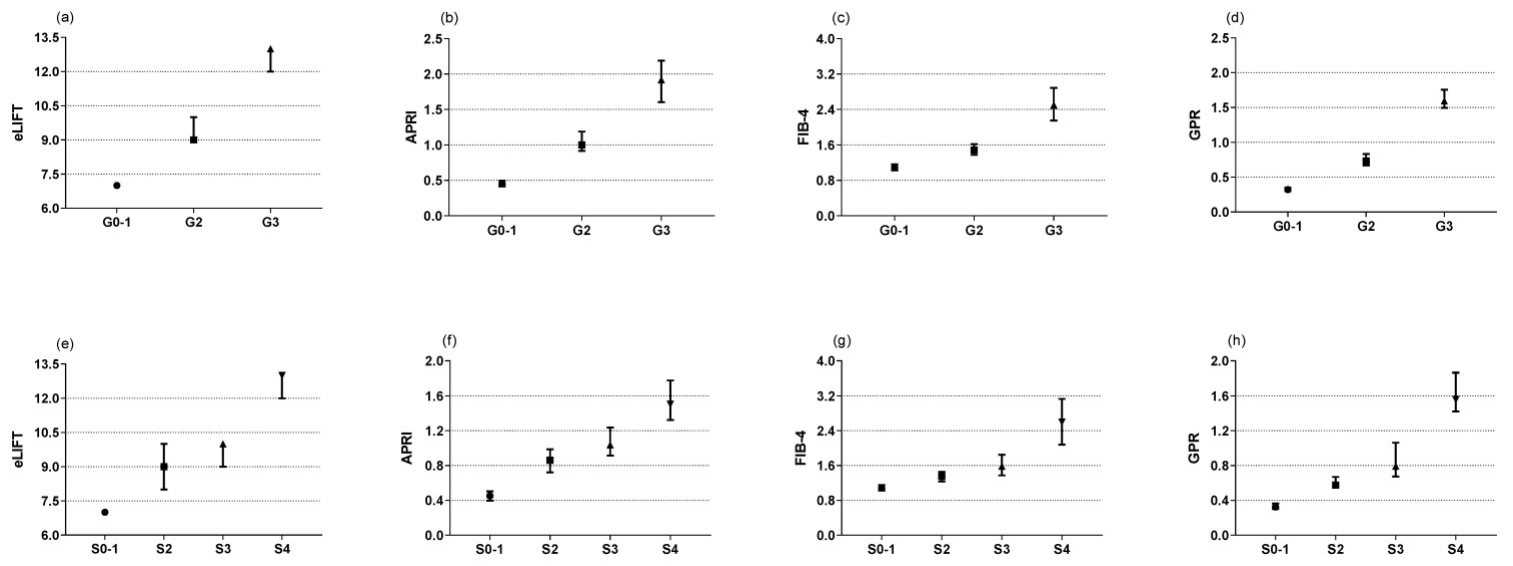
Medians in subgroups classified by inflammation grades and fibrosis stages (Scheuer scoring system). (a) the medians of eLIFT in G0-1, G2, and G3; (b) the medians of APRI in G0-1, G2, and G3; (c) the medians of FIB-4 in G0-1, G2, and G3; (d) the medians of GRP in G0-1, G2, and G3; (e) the medians of eLIFT in S0-1, S2, S3, and S4; (f) the medians of APRI in S0-1, S2, S3, and S4; (g) the medians of FIB-4 in S0-1, S2, S3, and S4; (h) the medians of GPR in S0-1, S2, S3, and S4.

The Kruskal-Wallis test showed that the eLIFT score, APRI, FIB-4, and GPR increased significantly in those with moderate to severe liver inflammation (G2-3) compared to G0-1 patients (**Figure 1a-d**). In regard to liver fibrosis, the same was true (**Figure 1e-f**). In other words, higher mode scores were seen with increasing liver histological scores. Spearman's correlation analysis showed that the eLIFT score (r = 0.517), APRI (r = 0.577), FIB-4 (r = 0.422), and GPR (r = 0.556) were significantly correlated with liver inflammatory activities. For liver fibrosis, the eLIFT score (r = 0.506), APRI (r = 0.487), FIB-4 (r = 0.395), and GPR (r = 0.507) were significantly correlated with fibrosis stage (**Table 2**).

### Performances of serum markers in the evaluation of liver inflammation

**Figure 2:**
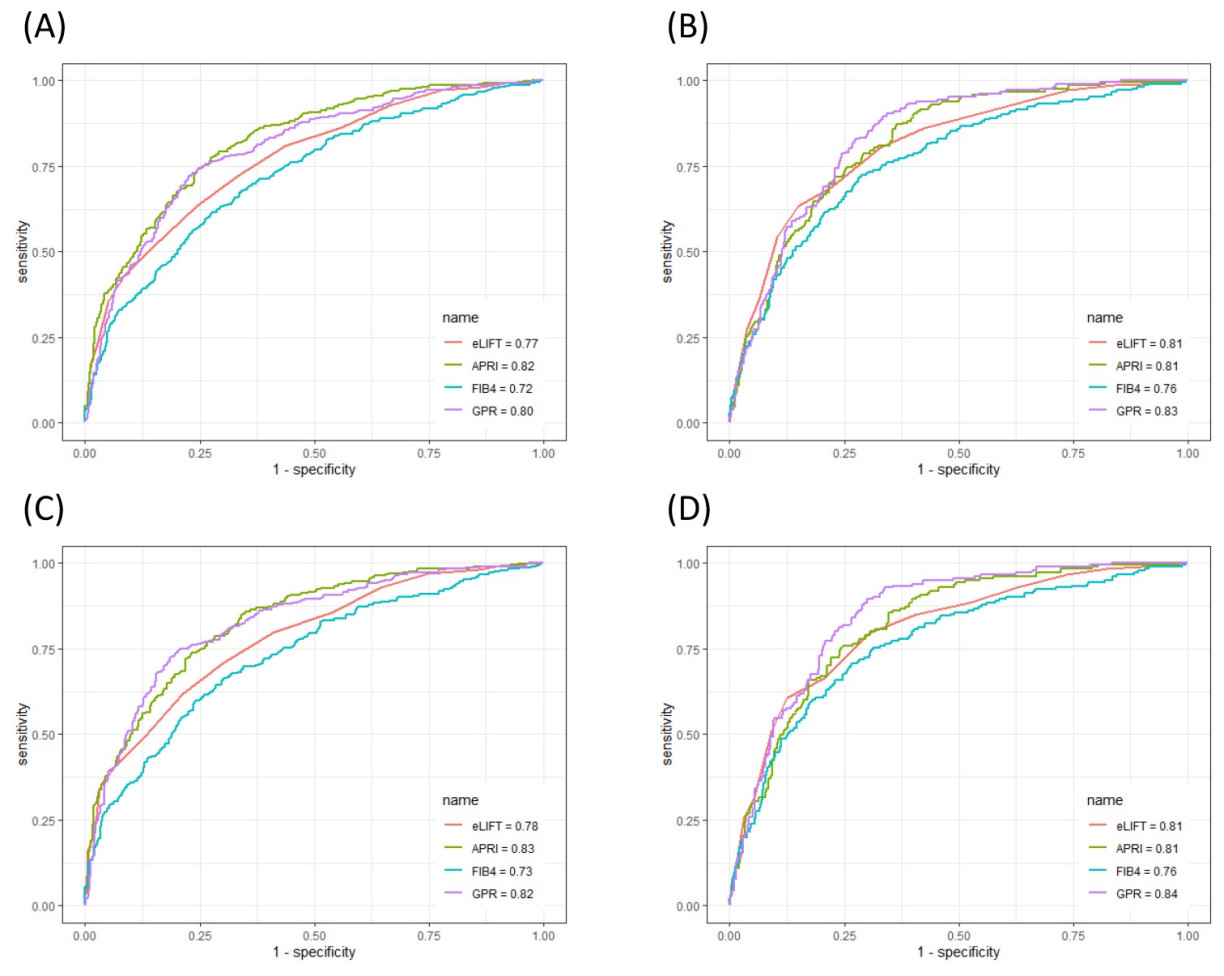
ROC comparison of eLIFT, APRI, FIB-4, and GPR for predicting liver inflammation. (A) ROC comparison for predicting G ≥ 2 in all patients; (B) ROC comparison for predicting G ≥ 3 in all patients; (C) ROC comparison for predicting G ≥ 2 in CHB patients; (D) ROC comparison for predicting G ≥ 3 in CHB patients.

The ROC curves of the eLIFT score, APRI, FIB-4, and GPR for predicting liver inflammation in all patients and in CHB patients are shown in **Figure 2**. In discriminating G≥2 inflammation, the AUROC of the eLIFT score was better than that of the FIB-4 (0.77 vs 0.72, respectively), with no difference compared with the GPR (0.77 vs 0.80, respectively), but lower than that of the APRI (0.77 vs 0.82, respectively). The optimal cut-off values for predicting G≥2 were 8.0 for the eLIFT score, 0.68 for the APRI, 1.48 for the FIB-4, and 0.58 for the GPR. When discriminating G≥3 inflammation, the AUROC of the eLIFT score was comparable to that of the APRI and GPR but superior to that of the FIB-4 (0.81, 0.81, 0.76, and 0.83, respectively) (**Table 3**).

Moreover, to investigate the influence of aetiology on the predictive performance of liver pathological scores, we further performed a subgroup analysis in the 853 enrolled CHB patients. In discriminating G≥2, the AUROCs of eLIFT, APRI, FIB-4, and GPR in CHB patients were 0.78, 0.83, 0.73, and 0.82, respectively. In discriminating G≥3, the AUROCs of the eLIFT score, APRI, FIB-4, and GPR were 0.81, 0.81, 0.76, and 0.84, respectively. Similarly, the AUROC of the eLIFT score for diagnosing liver inflammation was superior to that of the FIB-4 but lower than that of the APRI and GPR in CHB patients (**Table 4**).

### Performances of serum markers in the evaluation of liver fibrosis

**Figure 3:**
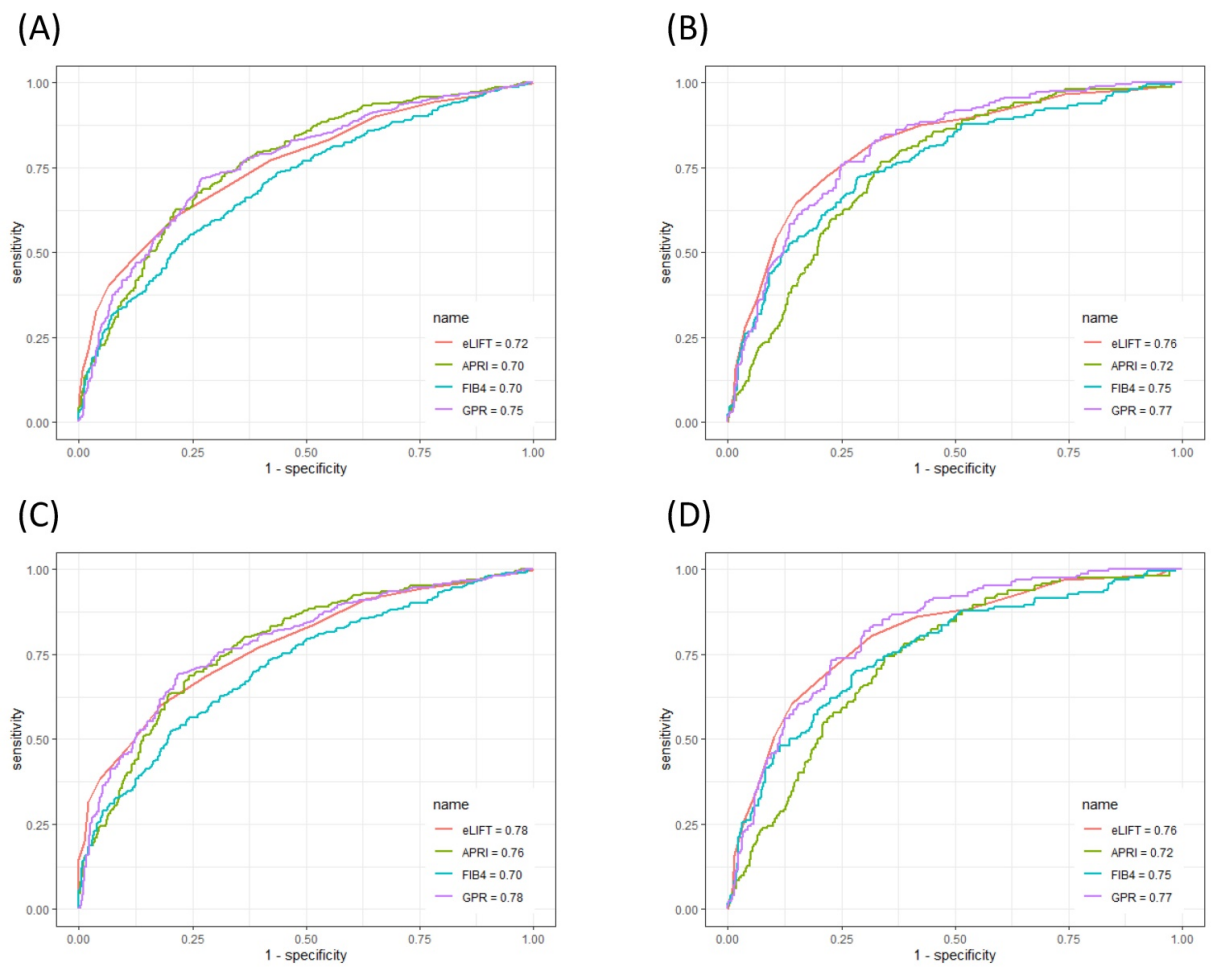
ROC comparison of eLIFT, APRI, FIB-4, and GPR for predicting liver fibrosis. (A) ROC comparison for predicting S ≥ 2 in all patients; (B) ROC comparison for predicting S4 in all patients; (C) ROC comparison for predicting S ≥ 2 in CHB patients; (B) ROC comparison for predicting S4 in CHB patients.

The ROC curves of the eLIFT score, APRI, FIB-4, and GPR for predicting liver inflammation in all patients and in CHB patients are shown in **Figure 3**. In discriminating S≥2 liver fibrosis, the AUROCs of the eLIFT score, APRI, FIB-4, and GPR were 0.72, 0.70, 0.70, and 0.75, respectively. The optimal cut-off values for predicting S≥2 were 9.0 for the eLIFT score, 0.76 for the APRI, 1.57 for the FIB-4, and 0.55 for the GPR. When discriminating S4, the AUROCs of the eLIFT score, APRI, FIB-4, and GPR were 0.76, 0.72, 0.75, and 0.77, respectively. The optimal cut-off values for predicting S4 were 10.0 for the eLIFT score, 0.85 for the APRI, 1.66 for the FIB-4, and 0.82 for the GPR. Interestingly, there were no significant differences among the four indexes for predicting S≥2 and S4 (**Table 5**).

In discriminating S≥2, the AUROCs of the eLIFT score, APRI, FIB-4, and GPR in CHB patients were 0.78, 0.76, 0.70, and 0.78, respectively. In discriminating S4, the AUROCs of the eLIFT score, APRI, FIB-4, and GPR were 0.76, 0.72, 0.75, and 0.77, respectively. Consistent with the results of the study in all patients, the eLIFT score was comparable to the APRI and GPR in predicting S≥2nd S4 in CHB patients (**Table 6**).

### DCA for clinical utility of the eLIFT

**Figure 4:**
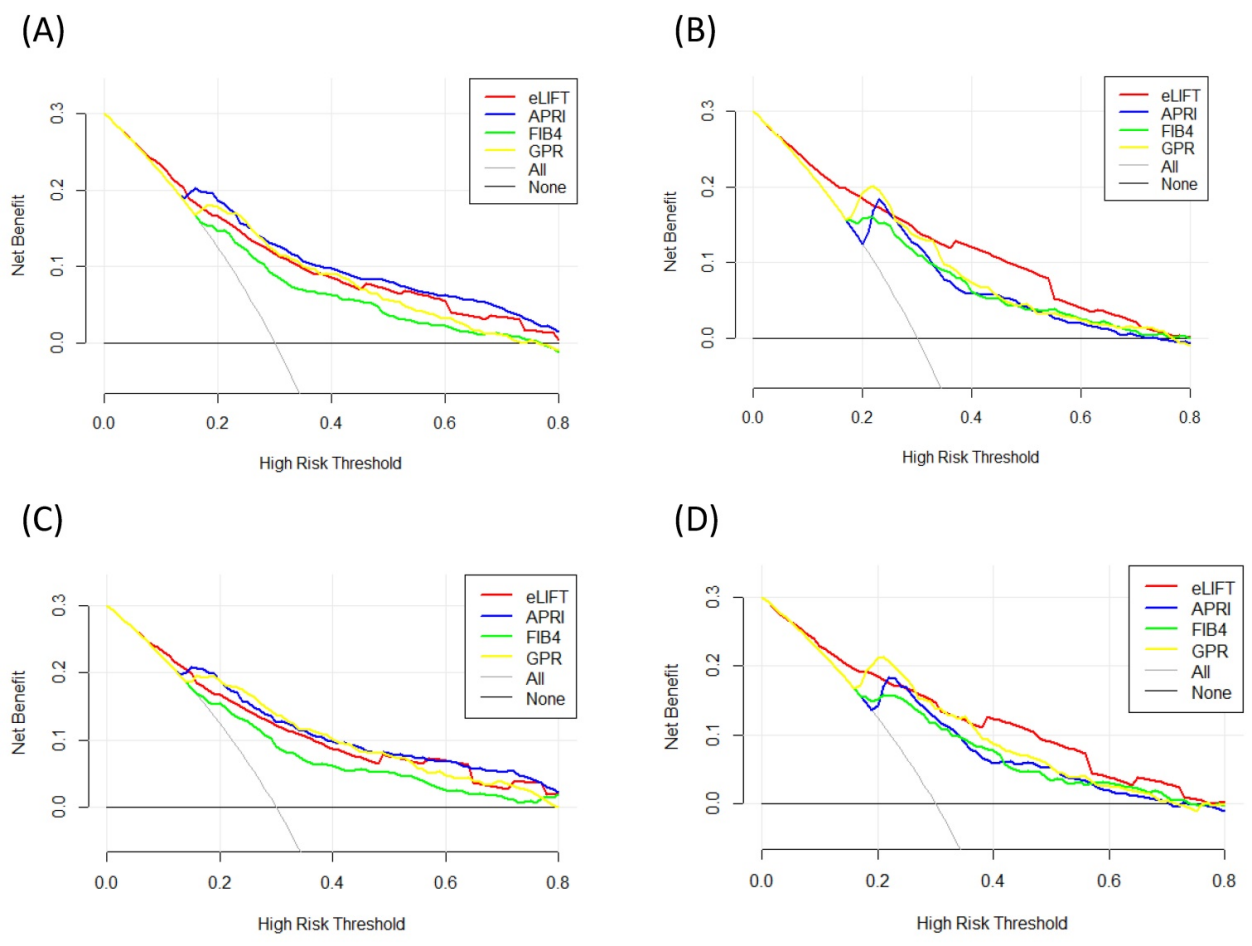
Liver fibrosis decision curve analysis. Decision curve analysis depict the clinical net benefit. The eLIFT was compared with APRI, FIB-4, and GPR for predicting liver inflammation. (A) for predicting G ≥ 2 in all patients; (B) for predicting G ≥ 3 in all patients; (C) for predicting G ≥ 2 in CHB patients; (D) for predicting G ≥ 3 in CHB patients. Black line = net benefit when no patient will experience the event; gray line = net benefit when all patients will experience the event. The preferred marker is the marker with the highest net benefit at any given threshold.

**Figure 5:**
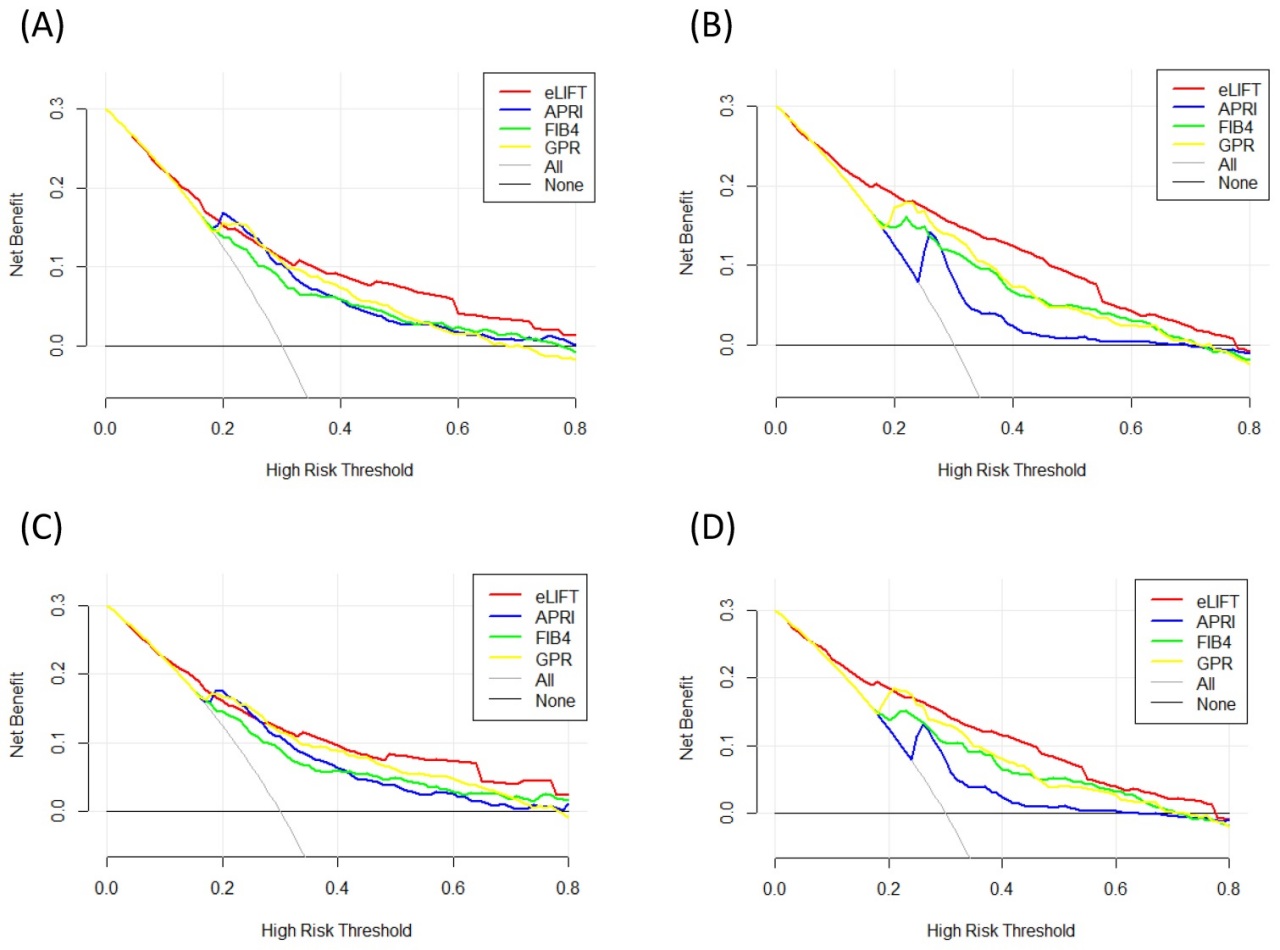
Liver fibrosis decision curve analysis. Decision curve analysis depict the clinical net benefit. The eLIFT was compared with APRI, FIB-4, and GPR for predicting liver fibrosis. (A) for predicting S ≥ 2 in all patients; (B) for predicting S4 in all patients; (C) for predicting S ≥ 2 in CHB patients; (D) for predicting S4 in CHB patients. Black line = net benefit when no patient will experience the event; gray line = net benefit when all patients will experience the event. The preferred markers is the marker with the highest net benefit at any given threshold.

We conducted DCA to further investigate the clinical application value of the eLIFT in predicting liver inflammation and fibrosis. DCA revealed that from a threshold probability of 10%-80%, application of the eLIFT to predict liver inflammation G≥3 in all patients and CHB patients was beneficial (**Figure 4**). Similarly, DCA also demonstrated that from a threshold probability of 10%-80%, application of the eLIFT to predict S≥2 and S4 liver fibrosis risk increased the benefit considerably more than the other three scores in all patients and in CHB patients (**Figure 5**).

## Discussion

In this retrospective cohort of CLD patients who underwent liver fibrosis, we validated the performances of the eLIFT, APRI, FIB-4 and GPR in diagnosing liver inflammation and fibrosis. Our study suggested that these markers might be potentially useful in predicting liver inflammation and fibrosis. The eLIFT and GPR might be useful serum indexes for evaluating the histological changes of CLD patients. The APRI, GPR, and eLIFT were superior to the FIB-4 for diagnosing liver inflammation. Similarly, the eLIFT and GPR were superior to the APRI and FIB-4 for diagnosing liver fibrosis.

The eLIFT is a new, user-friendly, rapid fibrosis test that can be used by all physicians who manage CLD patients in daily clinical practice, regardless of whether they specialize in hepatology, as it is based on the parameters usually evaluated in CLD 19. The eLIFT had two main advantages. First, compared with the APRI and FIB-4, which require a computer to calculate, the eLIFT score can be calculated very easily, which makes the eLIFT more convenient to use than the APRI and FIB-4 in clinical practice. Second, the eLIFT had fewer false positive results in 1251 CLD patients, so it was more suitable for screening 19. In this study, we confirmed the good clinical application value of the eLIFT. These results were consistent with previous studies 19, 21. The performance of the eLIFT was good for diagnosing liver inflammation, with AUROCs of 0.71 and 0.81 for G≥2 and G≥3, respectively. As expected, the performance of the eLIFT was also satisfactory for diagnosing liver fibrosis, with AUROCs of 0.73 and 0.76 for S≥2 and S4, respectively. Wang et al. 21 proposed that the eLIFT had similar diagnostic values for advanced fibrosis compared to more complex tools, such as the APRI, FIB-4, GPR, and RPR, in chronic hepatitis B (CHB) patients. However, in another larger study that included 747 CHB patients, the AUROC of the eLIFT was lower than those of the APRI and FIB-4 for diagnosing significant liver fibrosis and cirrhosis 22.

The GPR is a new noninvasive index for diagnosing liver fibrosis in CHB patients, although its diagnostic value is still controversial 17,23,24. A meta-analysis based on 10 studies reported that the AUROCs of the GPR for assessing significant fibrosis, advanced fibrosis, and cirrhosis were 0.73, 0.78, and 0.80, respectively 25. In our study, we found that the GRP was also satisfactory not only for the diagnosis of liver fibrosis but also for the diagnosis of liver inflammation. The GPR was comparable to the APRI but superior to the FIB-4 for predicting liver inflammation. The GPR exhibited AUROCs of 0.80 for G≥2, with an optimal cut-off of 0.58. The AUROC of the GPR increased to 0.83 when used to diagnose G≥3, with an optimal cut-off of 0.61. These results are consistent with previous studies 26, 27. Regarding liver fibrosis, the GPR was superior to the APRI and FIB-4 for diagnosing S≥2 and S4. The AUROCs of the GPR were 0.75 for S≥2 and 0.76 for S4. The GPR cut-off values (0.55 for S≥2, 0.82 for S4) were higher than those obtained by Lemoine 28 (0.32 for F≥2, 0.56 for F4).

One limitation of this study was that it was a single-centre retrospective study; thus, the results should be further confirmed in multicentre prospective studies with large-scale populations. Furthermore, since there were insufficient clinical data from FibroScan, we could not evaluate the performance of FibroScan for the diagnosis of liver inflammation and fibrosis.

In conclusion, the present study demonstrates that the eLIFT is a potentially useful noninvasive predictor of liver inflammation and fibrosis in patients with CLD. Although the eLIFT had similar diagnostic power to the liver histopathological score, compared with the APRI and GPR, in CLD patients, it had the advantage of easy implementation with wide clinical utility.

## Figures and Tables

**Table 1:** Clinical characteristics of the study patients

Variables	Total (n = 1125)	Inflammatory activity				Fibrosis stage		
		G0-1(n = 585)	G2-4(n = 540)	*P* value		S0-1 (n = 583)	S2-4 (n = 542)	*P* value
Age, years	37 (30-46)	37 (30-46)	36 (29-46)	0.196		37 (30-46)	36 (30-46)	0.544
Male, n (%)	734 (65.2)	392 (62.0)	342 (69.4)	0.010		351(61.4)	383 (69.3)	0.005
**Etiology of liver disease**								
HBV infection, n (%)	853 (75.8%)	440 (75.2%)	413 (76.5%)	0.620		434 (74.4%)	419 (77.3%)	0.262
HCV infection, n (%)	32 (2.8)	13 (2.2%)	19 (3.5%)	0.191		13 (2.2%)	19 (3.5%)	0.198
Non-alcoholic fatty liver disease, n (%)	144 (12.8%)	101 (17.3%)	43 (8.0%)	< 0.001		104 (17.8%)	40 (7.4%)	< 0.001
Alcoholic fatty liver disease, n (%)	43 (3.8%)	13 (2.2%)	30 (5.6%)	0.004		14 (2.4%)	29 (5.4%)	0.010
Autoimmune liver disease, n (%)	53 (4.7%)	18 (3.1%)	35 (6.5%)	0.007		18 (3.1%)	35 (6.5%)	0.008
**Serological parameters**								
ALT, U/L	59.0 (29.5-133.5)	38.0 (22.0-78.0)	101.0 (52.0-262.8)	<0.001		41.0 (22.0-87.0)	85.0 (41.0-185.0)	<0.001
AST, U/L	40.0 (25.0-83.5)	28.0 (21.0-44.0)	68.5 (40.0-141.0)	<0.001		29.0 (21.0-49.0)	57.0 (33.0-115.0)	<0.001
ALP, U/L	76.0 (63.0-97.0)	70.0 (57.0-84.0)	88.0 (72.0-113.0)	<0.001		71.0 (58.0-87.0)	84.0 (68.0-109.0)	<0.001
GGT, U/L	35.0 (19.0-78.0)	24.0 (16.0-41.0)	66.0 (35.0-118.5)	<0.001		24.0 (16.0-45.0)	57.0 (29.0-106.3)	<0.001
TBil, μmol/L	14.0 (10.0-19.8)	12.3 (9.3-16.7)	16.7 (11.4-25.0)	<0.001		12.3 (9.3-16.8)	15.9 (11.0-24.0)	<0.001
Albumin, g/L	42.3 (39.5-44.7)	43.1 (40.9-45.8)	40.8 (37.9-43.5)	<0.001		43.1(41.0-45.7)	41.0 (38.1-43.7)	<0.001
Globulin, g/L	28.0 (25.0-31.0)	27.0 (24.0-30.0)	29.0 (26.0-32.0)	<0.001		27.0 (24.0-30.0)	29.0 (26.0-32.0)	<0.001
Platelet, ×10^9^/L	160 (127-196)	172 (142-208)	144 (105-179)	<0.001		178 (149-210)	142 (108-176)	<0.001
Prothrombin time (%)	93 (85-102)	96 (88-104)	90 (80-99)	<0.001		98 (90-105)	89 (80-97)	<0.001
**Serological indexes**								
eLIFT	9.00 (6.00-11.00)	7.00 (5.00-9.00)	11.00 (8.00-13.00)	<0.001		7.00 (5.00-9.00)	10.00 (8.00-13.00)	<0.001
APRI	0.70 (0.36-1.53)	0.45 (0.29-0.77)	1.35 (0.75-2.92)	<0.001		0.45 (0.29-0.80)	1.14 (0.62-2.27)	<0.001
FIB-4	1.32 (0.90-2.19)	1.09 (0.77-1.58)	1.79 (1.19-3.42)	<0.001		1.08 (0.77-1.57)	1.69 (1.13-3.30)	<0.001
GPR	0.54 (0.26-1.26)	0.32 (0.19-0.58)	1.10 (0.57-2.06)	<0.001		0.32 (0.19-0.60)	0.92 (0.45-1.92)	<0.001

HBV, hepatitis B virus; HCV, hepatitis C virus; ALT, alanine aminotransferase; AST, aspartate aminotransferase; GGT, gamma-glutamyl transpeptidase; TBil, total bilirubin

**Table 2:** Correlation between the noninvasive indexes and liver pathology score

Indexes	Inflammatory activity			Fibrosis stage	
	r	*P* value		r	*P* value
eLIFT	0.517	<0.001		0.506	<0.001
APRI	0.577	<0.001		0.487	<0.001
FIB-4	0.422	<0.001		0.395	<0.001
GPR	0.556	<0.001		0.507	<0.001

**Table 3:** Predictive performance of serological indexes for assessing liver inflammatory in all patients (N = 1125)

	AUROC (95%CI)	P value	Cut-off	Se (%)	Sp (%)	PPV (%)	NPV (%)	Accuracy (%)	*P value
eLIFT									
G ≥ 2	0.77 (0.75-0.80)	<0.0001	8.0	72.1	66.8	65.5	73.1	69.2	--
G ≥ 3	0.81 (0.79-0.84)	<0.0001	11.0	63.3	85.2	52.4	90.0	67.5	--
APRI									
G ≥ 2	0.82 (0.80-0.84)	<0.0001	0.68	79.3	70.9	68.0	81.5	74.6	0.0001
G ≥ 3	0.81 (0.78-0.83)	<0.0001	0.78	87.1	63.7	35.5	95.6	65.3	0.798
FIB-4									
G ≥ 2	0.72 (0.69-0.75)	<0.0001	1.48	61.5	72.3	63.4	70.6	54.9	<0.0001
G ≥ 3	0.76 (0.73-0.79)	<0.0001	1.66	72.4	71.3	36.7	91.8	43.0	0.0001
GPR									
G ≥ 2	0.80 (0.77-0.82)	<0.0001	0.58	74.2	75.6	70.4	79.0	75.0	0.052
G ≥ 3	0.83 (0.81-0.85)	<0.0001	0.61	90.5	65.7	37.7	96.8	69.7	0.225

AUROC, area under ROC; Se, sensitivity; Sp, specificity; PPV, positive predictive value; NPV, negative predictive value. * Compared with eLIFT

**Table 4:** Predictive performance of serological indexes for assessing liver inflammatory in CHB patients (N = 853)

	AUROC (95%CI)	P value	Cut-off	Se (%)	Sp (%)	PPV (%)	NPV (%)	Accuracy (%)	*P value
eLIFT									
G ≥ 2	0.78 (0.75-0.80)	<0.0001	8.0	71.1	69.8	70.5	70.3	66.9	--
G ≥ 3	0.81 (0.78-0.84)	<0.0001	9.0	79.6	69.7	44.2	91.9	75.0	--
APRI									
G ≥ 2	0.83 (0.80-0.85)	<0.0001	0.55	85.9	65.1	68.8	83.7	74.7	0.001
G ≥ 3	0.81 (0.78-0.84)	<0.0001	1.04	75.7	75.3	45.2	92.0	75.3	0.898
FIB-4									
G ≥ 2	0.73 (0.69-0.76)	<0.0001	1.34	66.5	69.8	66.3	69.9	67.9	< 0.001
G ≥ 3	0.76 (0.73-0.80)	<0.0001	1.66	70.7	73.4	41.7	90.3	72.8	0.001
GPR									
G ≥ 2	0.82 (0.80-0.85)	<0.0001	0.55	74.9	79.3	76.5	77.9	77.3	0.004
G ≥ 3	0.84 (0.82-0.87)	<0.0001	0.61	89.5	69.9	44.5	96.1	74.0	0.038

AUROC, area under ROC; Se, sensitivity; Sp, specificity; PPV, positive predictive value; NPV, negative predictive value. * Compared with eLIFT

**Table 5:** Predictive performance of serological indexes for assessing liver fibrosis in all patients (N = 1125)

	AUROC (95%CI)	P value	Cut-off	Se (%)	Sp (%)	PPV (%)	NPV (%)	Accuracy	*P value
eLIFT									
S ≥ 2	0.72 (0.70-0.76)	<0.0001	9.0	60.6	78.7	76.8	63.1	62.7	--
S4	0.76 (0.72-0.80)	<0.0001	10.0	72.5	78.3	45.7	91.8	74.3	--
APRI									
S ≥ 2	0.70 (0.66-0.74)	<0.0001	0.76	67.7	73.9	71.5	70.4	70.1	0.448
S4	0.72 (0.67-0.75)	<0.0001	0.85	76.8	66.3	34.0	92.7	68.3	0.168
FIB-4									
S ≥ 2	0.70 (0.65-0.74)	<0.0001	1.57	55.3	75.4	68.5	63.6	65.5	0.359
S4	0.75 (0.71-0.79)	<0.0001	1.66	72.5	71.0	36.1	92.0	65.5	0.883
GPR									
S ≥ 2	0.75 (0.71-0.79)	<0.0001	0.55	71.8	72.9	71.9	72.8	72.4	0.272
S4	0.77 (0.73-0.80)	<0.0001	0.82	76.8	74.2	40.2	93.4	69.4	0.718

AUROC, area under ROC; Se, sensitivity; Sp, specificity; PPV, positive predictive value; NPV, negative predictive value. * Compared with eLIFT

**Table 6:** Predictive performance of serological indexes for assessing liver fibrosis in CHB patients (N = 853)

	AUROC (95%CI)	P value	Cut-off	Se (%)	Sp (%)	PPV (%)	NPV (%)	Accuracy	*P value
eLIFT									
S ≥ 2	0.78 (0.75-0.81)	<0.0001	9.0	60.1	81.9	81.0	61.5	66.2	--
S4	0.76 (0.72-0.80)	<0.0001	10.0	80.5	68.6	40.5	93.0	66.8	--
APRI									
S ≥ 2	0.76 (0.73-0.79)	<0.0001	0.76	68.6	75.9	73.4	69.7	71.7	0.398
S4	0.72 (0.67-0.75)	<0.0001	0.85	74.4	65.5	33.9	91.5	66.7	0.168
FIB-4									
S ≥ 2	0.70 (0.67-0.73)	<0.0001	1.48	56.3	75.9	71.2	62.1	65.3	< 0.0001
S4	0.75 (0.71-0.79)	<0.0001	1.65	70.1	71.8	37.2	91.0	71.4	0.883
GPR									
S ≥ 2	0.78 (0.75-0.81)	<0.0001	0.55	69.0	78.3	77.1	70.4	73.5	0.992
S4	0.77 (0.73-0.80)	<0.0001	0.67	81.7	70.0	39.3	94.1	72.1	0.718

AUROC, area under ROC; Se, sensitivity; Sp, specificity; PPV, positive predictive value; NPV, negative predictive value. * Compared with eLIFT
